# 1-{(*Z*)-[2-Meth­oxy-5-(trifluoro­meth­yl)anilino]methyl­idene}naphthalen-2(1*H*)-one

**DOI:** 10.1107/S1600536812051410

**Published:** 2013-01-04

**Authors:** Hakan Kargılı, Ayşen Alaman Ağar, Gökhan Alpaslan, Orhan Büyükgüngör, Ahmet Erdönmez

**Affiliations:** a Department of Physics, Faculty of Arts and Science, Ondokuz Mayıs University, TR-55139 Kurupelit-Samsun, Turkey; b Department of Chemistry, Faculty of Arts and Science, Ondokuz Mayıs University, TR-55139 Kurupelit-Samsun, Turkey; c Department of Medical Services and Techniques, Vocational School of Health Services, Giresun University, TR-28200 Giresun, Turkey

## Abstract

The title compound, C_19_H_14_F_3_NO_2_, crystallizes in the keto–amine tautomeric form, with a strong intra­molecular N—H⋯O hydrogen bond. The mol­ecule is almost planar; the dihedral angle between the naphthalene ring system and the benzene ring is 4.60 (7)°. In the crystal, mol­ecules are linked into chains along the *c* axis by C—H⋯O hydrogen bonds. The F atoms of the trifluoro­methyl group are disordered over two positions with refined site occupancies of 0.668 (9) and 0.332 (9).

## Related literature

For the biological properties of Schiff bases, see: Lozier *et al.* (1975 ▶). For the coordination chemistry of Schiff bases, see: Kargar *et al.* (2009 ▶); Yeap *et al.* (2009 ▶). For Schiff base tautomerism, see: Hökelek *et al.* (2000 ▶); Odabaşoğlu *et al.* (2005 ▶). For related strucures, see: Özek *et al.* (2004 ▶); Temel *et al.* (2010 ▶).
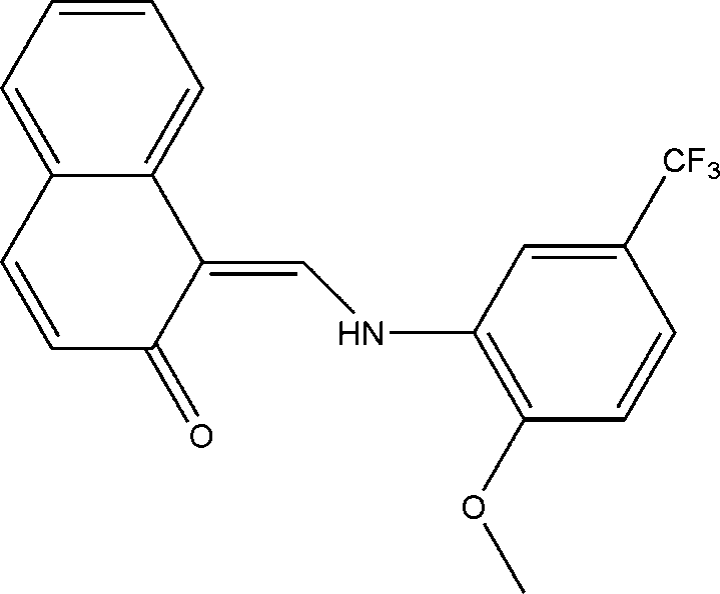


## Experimental

### 

#### Crystal data

C_19_H_14_F_3_NO_2_
*M* *_r_* = 345.31
Monoclinic, 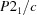

*a* = 16.3922 (10) Å
*b* = 5.7201 (2) Å
*c* = 17.7632 (10) Å
β = 106.284 (5)°
*V* = 1598.75 (14) Å^3^
*Z* = 4
Mo *K*α radiation
μ = 0.12 mm^−1^
*T* = 296 K
0.58 × 0.35 × 0.13 mm

#### Data collection

Stoe IPDSII diffractometer
Absorption correction: integration (*X-RED32*; Stoe & Cie, 2002 ▶) *T*_min_ = 0.949, *T*_max_ = 0.984
19794 measured reflections
3129 independent reflections
1722 reflections with *I* > 2σ(*I*)
*R*_int_ = 0.057

#### Refinement

*R*[*F* ^2^ > 2σ(*F* ^2^)] = 0.048
*wR*(*F* ^2^) = 0.097
*S* = 0.95
3129 reflections
258 parameters
72 restraints
H atoms treated by a mixture of independent and constrained refinement
Δρ_max_ = 0.15 e Å^−3^
Δρ_min_ = −0.17 e Å^−3^

### 

Data collection: *X-AREA* (Stoe & Cie, 2002 ▶); cell refinement: *X-AREA*; data reduction: *X-RED32* (Stoe & Cie, 2002 ▶); program(s) used to solve structure: *SHELXS97* (Sheldrick, 2008 ▶); program(s) used to refine structure: *SHELXL97* (Sheldrick, 2008 ▶); molecular graphics: *ORTEP-3 for Windows* (Farrugia, 2012 ▶); software used to prepare material for publication: *WinGX* (Farrugia, 2012 ▶).

## Supplementary Material

Crystal structure: contains datablock(s) I, global. DOI: 10.1107/S1600536812051410/fy2084sup1.cif

Structure factors: contains datablock(s) I. DOI: 10.1107/S1600536812051410/fy2084Isup2.hkl

Supplementary material file. DOI: 10.1107/S1600536812051410/fy2084Isup3.cml

Additional supplementary materials:  crystallographic information; 3D view; checkCIF report

## Figures and Tables

**Table 1:** Hydrogen-bond geometry (Å, °)

*D*—H⋯*A*	*D*—H	H⋯*A*	*D*⋯*A*	*D*—H⋯*A*
C8—H8⋯O1^i^	0.93	2.51	3.423 (3)	166
N1—H1⋯O1	1.00 (3)	1.70 (3)	2.554 (3)	140 (2)

Symmetry code: (i) 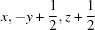.

**Fig. 1.**
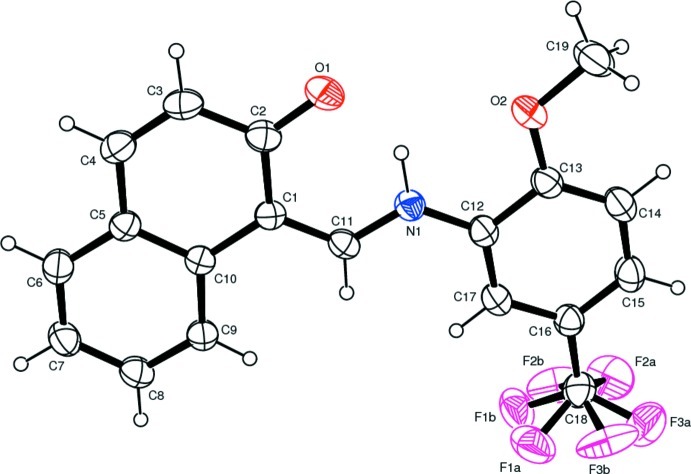
The molecular structure of (I), showing the atom-numbering scheme. Displacement ellipsoids are drawn at the 30% probability level.

**Fig. 2.**
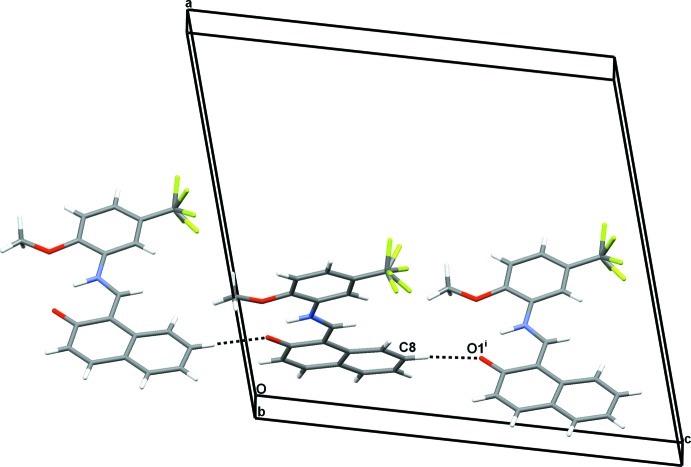
Packing diagram of the title compound; dashed lines indicate intermolecular hydrogen bonds.

## supplementary crystallographic information

### Comment

Schiff bases often exhibit various biological activities and in many cases were shown to have antibacterial, anticancer, anti-inflammatory and antitoxic properties (Lozier *et al.*, 1975). Schiff bases have also been used as versatile ligands in coordination chemistry (Kargar *et al.*, 2009; Yeap *et al.*, 2009). There are two types of intramolecular hydrogen bonds in Schiff bases, namely N—H···O in keto (NH) (Hökelek *et al.*, 2000) and N···H—O in enol (OH) (Odabaşoǧlu *et al.*, 2005) tautomeric forms. The present X-ray investigation shows that the title compound is a Schiff base and exists in the keto-amine form in the solid state. An ORTEP-3 (Farrugia, 2012) plot of the molecule of (I) is shown in Fig. 1. The C2—O1 bond length of 1.272 (3) Å indicates double-bond character while the N1—C11 bond length of 1.322 (3) Å indicates single-bond character. These bond distances are comparable with those of compounds previously reported as keto–amine (Özek *et al.*, 2004; Temel *et al.*, 2010). The dihedral angle between the naphthalene ring system and the benzene ring is 4.60 (7)°. In the crystal, molecules are linked into chains (Fig. 2) along the *c* axis by intermolecular C-H···O hydrogen bonds (Table 1).

### Experimental

(Z)-1-[(2-Methoxy-5-(trifluoromethyl)phenylamino)methylene]naphthalen-2(1H)-one was prepared by refluxing a mixture of a solution containing 2-hydroxy-1-naphthaldehyde (17.22 mg, 0.1 mmol) in ethanol (20 ml) and a solution containing 2-methoxy-5-(trifluoromethyl)aniline (19.12 mg, 0.1 mmol) in ethanol (20 ml). The reaction mixture was stirred for 5 h under reflux. Single crystals of the title compound for X-ray analysis were obtained by slow evaporation of an ethanol solution (Yield 84%; m.p. 472–475 K).

### Refinement

All H atoms (except for H1) were placed in calculated positions and constrained to ride on their parent atoms, with C–H = 0.93-0.96 Å and *U*_iso_(H) = 1.2 *U*_eq_(C)[1.5*U*_eq_(C)for the methyl H atoms]. The CF_3_ group showed rotational disorder. For atoms F1A, F2A and F3A the site occupancy factor is 0.668 (9) and for F1B, F2B and F3B the site occupancy factor is 0.332 (9). The disorder was refined using the commands DFIX, ISOR and SIMU. DFIX was used to restrain all C—F bond lengths to 1.322 (15) Å, while the ISOR and SIMU restraints were applied for all F atoms with effective standard deviation values of 0.01 and 0.04 Å^2^, respectively.

### Crystal data

**Table:**

C_19_H_14_F_3_NO_2_	*F*(000) = 712
*M**_r_* = 345.31	*D*_x_ = 1.435 Mg m^−^^3^
Monoclinic, *P*2_1_/*c*	Mo *K*α radiation, λ = 0.71073 Å
Hall symbol: -P 2ybc	Cell parameters from 15281 reflections
*a* = 16.3922 (10) Å	θ = 1.5–28.0°
*b* = 5.7201 (2) Å	µ = 0.12 mm^−^^1^
*c* = 17.7632 (10) Å	*T* = 296 K
β = 106.284 (5)°	Prism, brown
*V* = 1598.75 (14) Å^3^	0.58 × 0.35 × 0.13 mm
*Z* = 4	

### Data collection

**Table:**

Stoe IPDSII diffractometer	3129 independent reflections
Radiation source: fine-focus sealed tube	1722 reflections with *I* > 2σ(*I*)
Graphite monochromator	*R*_int_ = 0.057
Detector resolution: 6.67 pixels mm^-1^	θ_max_ = 26.0°, θ_min_ = 2.4°
ω scans	*h* = −20→20
Absorption correction: integration (*X-RED32*; Stoe & Cie, 2002)	*k* = −7→7
*T*_min_ = 0.949, *T*_max_ = 0.984	*l* = −21→21
19794 measured reflections	

### Refinement

**Table:**

Refinement on *F*^2^	Primary atom site location: structure-invariant direct methods
Least-squares matrix: full	Secondary atom site location: difference Fourier map
*R*[*F*^2^ > 2σ(*F*^2^)] = 0.048	Hydrogen site location: inferred from neighbouring sites
*wR*(*F*^2^) = 0.097	H atoms treated by a mixture of independent and constrained refinement
*S* = 0.95	*w* = 1/[σ^2^(*F*_o_^2^) + (0.0399*P*)^2^] where *P* = (*F*_o_^2^ + 2*F*_c_^2^)/3
3129 reflections	(Δ/σ)_max_ < 0.001
258 parameters	Δρ_max_ = 0.15 e Å^−^^3^
72 restraints	Δρ_min_ = −0.17 e Å^−^^3^

### Special details

**Table:**

Geometry. All esds (except the esd in the dihedral angle between two l.s. planes) are estimated using the full covariance matrix. The cell esds are taken into account individually in the estimation of esds in distances, angles and torsion angles; correlations between esds in cell parameters are only used when they are defined by crystal symmetry. An approximate (isotropic) treatment of cell esds is used for estimating esds involving l.s. planes.
Refinement. Refinement of F^2^ against ALL reflections. The weighted R-factor wR and goodness of fit S are based on F^2^, conventional R-factors R are based on F, with F set to zero for negative F^2^. The threshold expression of F^2^ > 2sigma(F^2^) is used only for calculating R-factors(gt) etc. and is not relevant to the choice of reflections for refinement. R-factors based on F^2^ are statistically about twice as large as those based on F, and R- factors based on ALL data will be even larger.

### Fractional atomic coordinates and isotropic or equivalent isotropic displacement parameters (Å^2^)

**Table:**

	*x*	*y*	*z*	*U*_iso_*/*U*_eq_	Occ. (<1)
C1	0.17645 (13)	0.2354 (4)	0.18859 (12)	0.0443 (6)	
C2	0.15233 (15)	0.1866 (5)	0.10590 (13)	0.0527 (6)	
C3	0.10170 (16)	−0.0181 (5)	0.07934 (13)	0.0589 (7)	
H3	0.0853	−0.0530	0.0261	0.071*	
C4	0.07736 (15)	−0.1601 (5)	0.12881 (13)	0.0573 (7)	
H4	0.0450	−0.2913	0.1088	0.069*	
C5	0.09943 (13)	−0.1173 (4)	0.21140 (13)	0.0459 (6)	
C6	0.07423 (15)	−0.2706 (4)	0.26206 (14)	0.0568 (6)	
H6	0.0436	−0.4041	0.2417	0.068*	
C7	0.09358 (16)	−0.2288 (5)	0.34056 (14)	0.0625 (7)	
H7	0.0765	−0.3326	0.3735	0.075*	
C8	0.13909 (17)	−0.0297 (5)	0.37078 (13)	0.0611 (7)	
H8	0.1519	0.0010	0.4242	0.073*	
C9	0.16550 (15)	0.1225 (4)	0.32305 (12)	0.0543 (6)	
H9	0.1960	0.2550	0.3447	0.065*	
C10	0.14750 (13)	0.0831 (4)	0.24208 (12)	0.0424 (5)	
C11	0.22965 (14)	0.4238 (4)	0.21675 (13)	0.0466 (6)	
H11	0.2449	0.4530	0.2704	0.056*	
C12	0.31391 (14)	0.7553 (4)	0.19682 (13)	0.0470 (6)	
C13	0.33425 (15)	0.8888 (4)	0.13842 (13)	0.0518 (6)	
C14	0.38600 (16)	1.0818 (5)	0.15828 (15)	0.0611 (7)	
H14	0.3995	1.1696	0.1194	0.073*	
C15	0.41778 (15)	1.1448 (5)	0.23597 (16)	0.0616 (7)	
H15	0.4525	1.2757	0.2492	0.074*	
C16	0.39861 (15)	1.0159 (4)	0.29414 (14)	0.0526 (6)	
C17	0.34681 (14)	0.8209 (4)	0.27413 (13)	0.0531 (6)	
H17	0.3341	0.7330	0.3134	0.064*	
C18	0.43282 (19)	1.0816 (6)	0.37746 (17)	0.0706 (8)	
C19	0.3127 (2)	0.9460 (5)	0.00083 (15)	0.0800 (9)	
H19A	0.2853	0.8708	−0.0480	0.120*	
H19B	0.3725	0.9578	0.0066	0.120*	
H19C	0.2893	1.0997	0.0014	0.120*	
F1A	0.3844 (4)	1.0304 (15)	0.4230 (2)	0.112 (2)	0.668 (9)
F2A	0.5162 (2)	1.0948 (14)	0.3989 (3)	0.1156 (18)	0.668 (9)
F3A	0.4413 (6)	1.3154 (9)	0.3887 (3)	0.139 (2)	0.668 (9)
F1B	0.4342 (10)	0.897 (2)	0.4242 (4)	0.108 (4)	0.332 (9)
F2B	0.4937 (8)	0.945 (3)	0.4172 (5)	0.113 (4)	0.332 (9)
F3B	0.3934 (8)	1.252 (3)	0.3936 (6)	0.126 (5)	0.332 (9)
N1	0.25997 (12)	0.5641 (3)	0.17194 (11)	0.0489 (5)	
O1	0.17545 (12)	0.3149 (3)	0.05715 (9)	0.0707 (5)	
O2	0.29915 (11)	0.8115 (3)	0.06414 (9)	0.0651 (5)	
H1	0.2375 (18)	0.521 (5)	0.1153 (17)	0.100 (10)*	

### Atomic displacement parameters (Å^2^)

**Table:**

	*U*^11^	*U*^22^	*U*^33^	*U*^12^	*U*^13^	*U*^23^
C1	0.0468 (13)	0.0434 (15)	0.0421 (12)	0.0046 (11)	0.0116 (10)	0.0010 (11)
C2	0.0547 (14)	0.0570 (16)	0.0451 (13)	0.0073 (13)	0.0120 (11)	0.0037 (13)
C3	0.0589 (16)	0.0727 (19)	0.0403 (12)	0.0039 (14)	0.0064 (11)	−0.0073 (13)
C4	0.0540 (15)	0.0602 (17)	0.0557 (15)	−0.0027 (13)	0.0117 (12)	−0.0125 (13)
C5	0.0426 (13)	0.0439 (14)	0.0516 (13)	0.0045 (11)	0.0141 (10)	−0.0030 (11)
C6	0.0534 (15)	0.0492 (16)	0.0699 (16)	−0.0039 (12)	0.0207 (13)	−0.0024 (13)
C7	0.0706 (17)	0.0593 (18)	0.0631 (16)	−0.0044 (15)	0.0281 (13)	0.0099 (14)
C8	0.0710 (17)	0.0699 (19)	0.0436 (13)	−0.0054 (15)	0.0179 (12)	0.0046 (13)
C9	0.0596 (15)	0.0574 (17)	0.0458 (13)	−0.0090 (13)	0.0144 (11)	−0.0018 (12)
C10	0.0391 (12)	0.0447 (14)	0.0431 (12)	0.0036 (11)	0.0113 (9)	0.0015 (11)
C11	0.0485 (13)	0.0489 (15)	0.0447 (12)	0.0066 (12)	0.0167 (11)	0.0070 (12)
C12	0.0470 (13)	0.0420 (14)	0.0551 (14)	0.0081 (12)	0.0195 (11)	0.0077 (12)
C13	0.0521 (14)	0.0543 (17)	0.0534 (15)	0.0044 (12)	0.0221 (12)	0.0049 (12)
C14	0.0652 (16)	0.0610 (18)	0.0643 (17)	−0.0004 (15)	0.0298 (13)	0.0125 (14)
C15	0.0542 (16)	0.0566 (17)	0.0786 (18)	−0.0081 (13)	0.0259 (14)	0.0024 (15)
C16	0.0476 (14)	0.0523 (16)	0.0598 (15)	−0.0011 (12)	0.0180 (11)	0.0005 (13)
C17	0.0549 (14)	0.0540 (16)	0.0538 (14)	0.0009 (13)	0.0206 (12)	0.0088 (12)
C18	0.065 (2)	0.070 (2)	0.077 (2)	−0.0131 (18)	0.0216 (16)	−0.0105 (18)
C19	0.099 (2)	0.090 (2)	0.0601 (16)	−0.0002 (18)	0.0382 (15)	0.0151 (16)
F1A	0.114 (3)	0.164 (5)	0.0654 (19)	−0.055 (3)	0.036 (2)	−0.023 (2)
F2A	0.072 (2)	0.171 (5)	0.091 (2)	−0.037 (3)	0.0024 (16)	−0.023 (3)
F3A	0.222 (6)	0.094 (3)	0.104 (3)	−0.050 (4)	0.048 (4)	−0.029 (2)
F1B	0.144 (8)	0.118 (6)	0.058 (3)	−0.058 (6)	0.024 (5)	0.008 (4)
F2B	0.086 (6)	0.154 (8)	0.080 (5)	0.052 (6)	−0.006 (4)	−0.029 (5)
F3B	0.129 (7)	0.140 (9)	0.100 (5)	0.047 (6)	0.018 (5)	−0.061 (6)
N1	0.0534 (12)	0.0474 (13)	0.0470 (11)	0.0008 (10)	0.0156 (9)	0.0033 (10)
O1	0.0933 (14)	0.0756 (13)	0.0442 (9)	−0.0021 (11)	0.0208 (9)	0.0103 (9)
O2	0.0776 (12)	0.0706 (13)	0.0525 (10)	−0.0053 (10)	0.0273 (9)	0.0083 (9)

### Geometric parameters (Å, º)

**Table:**

C1—C11	1.388 (3)	C12—C13	1.402 (3)
C1—C2	1.437 (3)	C13—O2	1.357 (3)
C1—C10	1.463 (3)	C13—C14	1.377 (3)
C2—O1	1.272 (3)	C14—C15	1.379 (3)
C2—C3	1.436 (3)	C14—H14	0.9300
C3—C4	1.337 (3)	C15—C16	1.376 (3)
C3—H3	0.9300	C15—H15	0.9300
C4—C5	1.430 (3)	C16—C17	1.387 (3)
C4—H4	0.9300	C16—C18	1.477 (4)
C5—C6	1.399 (3)	C17—H17	0.9300
C5—C10	1.411 (3)	C18—F3B	1.248 (8)
C6—C7	1.362 (3)	C18—F2B	1.308 (8)
C6—H6	0.9300	C18—F1A	1.315 (4)
C7—C8	1.385 (3)	C18—F2A	1.315 (4)
C7—H7	0.9300	C18—F1B	1.339 (8)
C8—C9	1.367 (3)	C18—F3A	1.354 (5)
C8—H8	0.9300	C19—O2	1.430 (3)
C9—C10	1.403 (3)	C19—H19A	0.9600
C9—H9	0.9300	C19—H19B	0.9600
C11—N1	1.322 (3)	C19—H19C	0.9600
C11—H11	0.9300	F2A—F3A	1.735 (10)
C12—C17	1.379 (3)	F1B—F2B	1.05 (2)
C12—N1	1.398 (3)	N1—H1	1.00 (3)
			
C11—C1—C2	119.0 (2)	C13—C14—C15	119.9 (2)
C11—C1—C10	120.90 (19)	C13—C14—H14	120.0
C2—C1—C10	120.1 (2)	C15—C14—H14	120.0
O1—C2—C3	120.1 (2)	C16—C15—C14	120.7 (2)
O1—C2—C1	122.3 (2)	C16—C15—H15	119.7
C3—C2—C1	117.5 (2)	C14—C15—H15	119.7
C4—C3—C2	122.1 (2)	C15—C16—C17	119.4 (2)
C4—C3—H3	119.0	C15—C16—C18	120.9 (3)
C2—C3—H3	119.0	C17—C16—C18	119.7 (2)
C3—C4—C5	122.3 (2)	C12—C17—C16	121.0 (2)
C3—C4—H4	118.9	C12—C17—H17	119.5
C5—C4—H4	118.9	C16—C17—H17	119.5
C6—C5—C10	119.6 (2)	F3B—C18—F2B	135.2 (6)
C6—C5—C4	121.1 (2)	F1A—C18—F2A	126.4 (4)
C10—C5—C4	119.2 (2)	F3B—C18—F1B	113.3 (7)
C7—C6—C5	121.5 (2)	F2B—C18—F1B	46.8 (11)
C7—C6—H6	119.3	F1A—C18—F3A	100.6 (4)
C5—C6—H6	119.3	F2A—C18—F3A	81.1 (6)
C6—C7—C8	119.1 (2)	F3B—C18—C16	110.7 (5)
C6—C7—H7	120.4	F2B—C18—C16	114.0 (4)
C8—C7—H7	120.4	F1A—C18—C16	115.8 (3)
C9—C8—C7	120.9 (2)	F2A—C18—C16	112.1 (3)
C9—C8—H8	119.6	F1B—C18—C16	110.9 (4)
C7—C8—H8	119.6	F3A—C18—C16	113.2 (4)
C8—C9—C10	121.4 (2)	O2—C19—H19A	109.5
C8—C9—H9	119.3	O2—C19—H19B	109.5
C10—C9—H9	119.3	H19A—C19—H19B	109.5
C9—C10—C5	117.4 (2)	O2—C19—H19C	109.5
C9—C10—C1	123.9 (2)	H19A—C19—H19C	109.5
C5—C10—C1	118.73 (19)	H19B—C19—H19C	109.5
N1—C11—C1	124.0 (2)	C18—F2A—F3A	50.4 (3)
N1—C11—H11	118.0	C18—F3A—F2A	48.5 (3)
C1—C11—H11	118.0	F2B—F1B—C18	65.0 (8)
C17—C12—N1	124.3 (2)	F1B—F2B—C18	68.1 (8)
C17—C12—C13	118.8 (2)	C11—N1—C12	126.6 (2)
N1—C12—C13	116.9 (2)	C11—N1—H1	111.3 (17)
O2—C13—C14	125.0 (2)	C12—N1—H1	122.1 (17)
O2—C13—C12	114.7 (2)	C13—O2—C19	118.2 (2)
C14—C13—C12	120.3 (2)		
			
C11—C1—C2—O1	−2.7 (3)	C18—C16—C17—C12	180.0 (2)
C10—C1—C2—O1	179.5 (2)	C15—C16—C18—F3B	76.5 (10)
C11—C1—C2—C3	176.1 (2)	C17—C16—C18—F3B	−103.8 (10)
C10—C1—C2—C3	−1.7 (3)	C15—C16—C18—F2B	−106.0 (11)
O1—C2—C3—C4	178.8 (2)	C17—C16—C18—F2B	73.6 (11)
C1—C2—C3—C4	−0.1 (3)	C15—C16—C18—F1A	149.0 (5)
C2—C3—C4—C5	0.5 (4)	C17—C16—C18—F1A	−31.4 (6)
C3—C4—C5—C6	−179.0 (2)	C15—C16—C18—F2A	−55.9 (5)
C3—C4—C5—C10	0.9 (3)	C17—C16—C18—F2A	123.7 (5)
C10—C5—C6—C7	1.3 (3)	C15—C16—C18—F1B	−156.9 (9)
C4—C5—C6—C7	−178.7 (2)	C17—C16—C18—F1B	22.8 (10)
C5—C6—C7—C8	0.1 (4)	C15—C16—C18—F3A	33.6 (6)
C6—C7—C8—C9	−0.8 (4)	C17—C16—C18—F3A	−146.8 (5)
C7—C8—C9—C10	0.1 (4)	F3B—C18—F2A—F3A	−17.8 (9)
C8—C9—C10—C5	1.3 (3)	F2B—C18—F2A—F3A	−145.9 (6)
C8—C9—C10—C1	−177.9 (2)	F1A—C18—F2A—F3A	−96.6 (6)
C6—C5—C10—C9	−2.0 (3)	F1B—C18—F2A—F3A	−135.1 (6)
C4—C5—C10—C9	178.0 (2)	C16—C18—F2A—F3A	111.5 (4)
C6—C5—C10—C1	177.3 (2)	F3B—C18—F3A—F2A	154.8 (11)
C4—C5—C10—C1	−2.7 (3)	F2B—C18—F3A—F2A	27.3 (7)
C11—C1—C10—C9	4.5 (3)	F1A—C18—F3A—F2A	125.6 (4)
C2—C1—C10—C9	−177.7 (2)	F1B—C18—F3A—F2A	83.5 (10)
C11—C1—C10—C5	−174.70 (19)	C16—C18—F3A—F2A	−110.3 (4)
C2—C1—C10—C5	3.1 (3)	F3B—C18—F1B—F2B	−131.0 (8)
C2—C1—C11—N1	0.2 (3)	F1A—C18—F1B—F2B	−149.5 (8)
C10—C1—C11—N1	178.1 (2)	F2A—C18—F1B—F2B	−10.6 (7)
C17—C12—C13—O2	180.0 (2)	F3A—C18—F1B—F2B	−89.7 (11)
N1—C12—C13—O2	−0.8 (3)	C16—C18—F1B—F2B	103.9 (6)
C17—C12—C13—C14	−0.1 (3)	F3B—C18—F2B—F1B	79.9 (17)
N1—C12—C13—C14	179.0 (2)	F1A—C18—F2B—F1B	22.7 (7)
O2—C13—C14—C15	179.6 (2)	F2A—C18—F2B—F1B	165.1 (10)
C12—C13—C14—C15	−0.2 (4)	F3A—C18—F2B—F1B	126.0 (8)
C13—C14—C15—C16	0.3 (4)	C16—C18—F2B—F1B	−96.7 (8)
C14—C15—C16—C17	0.0 (4)	C1—C11—N1—C12	−179.7 (2)
C14—C15—C16—C18	179.6 (2)	C17—C12—N1—C11	2.8 (3)
N1—C12—C17—C16	−178.7 (2)	C13—C12—N1—C11	−176.3 (2)
C13—C12—C17—C16	0.4 (3)	C14—C13—O2—C19	−3.5 (3)
C15—C16—C17—C12	−0.4 (3)	C12—C13—O2—C19	176.4 (2)

### Hydrogen-bond geometry (Å, º)

**Table:**

*D*—H···*A*	*D*—H	H···*A*	*D*···*A*	*D*—H···*A*
C8—H8···O1^i^	0.93	2.51	3.423 (3)	166
N1—H1···O1	1.00 (3)	1.70 (3)	2.554 (3)	140 (2)

Symmetry code: (i) *x*, −*y*+1/2, *z*+1/2.
